# IgA nephropathy in children: how much can we trust prognostic tools?

**DOI:** 10.1590/2175-8239-JBN-2025-E012en

**Published:** 2025-09-08

**Authors:** Maria Goretti Moreira Guimarães Penido, Nilzete Liberato Bresolin, Oreste Ferra-Neto

**Affiliations:** 1 Hospital Santa Casa de Belo Horizonte, Centro de Nefrologia, Unidade de Nefrologia Pediátrica, Belo Horizonte, MG, Brazil.; 2 Universidade Federal de Minas Gerais, Faculdade de Medicina, Hospital das Clínicas, Unidade de Nefrologia Pediátrica, Belo Horizonte, MG, Brazil.; 3 Universidade Federal de Santa Catarina, Faculdade de Medicina, Florianópolis, SC, Brazil.; 4 Hospital Infantil Joana de Gusmão, Unidade de Nefrologia Pediátrica e Terapia Intensiva, Florianópolis, SC, Brazil.; 5 Universidade Federal do Mato Grosso do Sul, Hospital Universitário Maria Aparecida Pedrossian, Unidade de Nefrologia Pediátrica, Campo Grande, MS, Brazil.

Immunoglobulin A deposition nephropathy (IgAN) results from deposits of IgA1-IgG immune complexes in the glomeruli, promoting an inflammatory cascade that leads to renal injury^1,2^. It is the most common primary glomerulonephritis worldwide and its definitive diagnosis is made by renal biopsy^3,4,5^.

The International Pediatric Nephrology Association (IPNA) has issued comprehensive, evidence-based clinical practice recommendations for the diagnosis and management of children with IgAN. The guidelines highlight key differences between pediatric and adult IgAN, as recently confirmed by a large study in the Chinese population^2,6^. Children often present with gross hematuria during infections (synpharyngitic hematuria), and kidney biopsies typically show more active inflammation with less chronic damage than adults^6^. A kidney biopsy is strongly recommended in cases of persistent or recurrent hematuria associated with significant proteinuria (UPCR ≥ 0.5 mg/mg), nephrotic-range proteinuria (UPCR ≥ 2 mg/mg), or reduced eGFR (< 90 mL/min/1.73 m^2^). Biopsy is also required in patients with persistent microscopic hematuria and UPCR between 0.2–0.5 mg/mg over several assessments.

The disease rarely progresses to stage 5 chronic kidney disease (CKD) in childhood^7^, but it is a chronic disease and pediatric patients are at risk of progression throughout adulthood, although sponta-neous remissions are possible in mild cases^8^. Thus, pediatric IgAN is recognized as a disease requiring specialized care^9^.

Supportive care remains the corner-stone of treatment: renin-angiotensin system blockers (RASB) are recommended for persistent proteinuria (UPCR ≥ 0.2 mg/mg), along with dietary salt restriction and lifestyle measures. For children with CKD higher than or equal stage 2, tighter blood pressure targets are advised. Glucocorticoids are not routinely advised but may be considered in selected cases with persistent proteinuria, after 3–6 months of optimized supportive therapy in patients with persistent proteinuria ≥ 1 mg/mg or high-risk biopsy findings. It is important to note that, the guideline warns against immunosuppressive use without histologic confirmation.^2^

Long-term follow-up is emphasized for all patients—even those in remission—due to the risk of late relapse and progression to CKD^2^.

Considering that the management of pediatric IgAN is still controversial and supported by little high-quality evidence, and that the scarcity of evidence is related to the heterogeneity of the disease in children and adolescents and its interethnic variability, the study by Cabral et al., published in the Brazilian Journal of Nephrology deserves some observations and considerations based on recent publications.

## Results of Cabral MF et al. Study

Cabral et al.^10^ retrospectively studied 23 children with IgAN. They compared the actual renal outcomes with outcomes predicted by the International IgAN Prediction Tool for Children (IIgAN-PT)^11,12^. The primary outcome was a composite outcome of ≥30% reduction in eGFR or progression to end-stage kidney disease (ESKD). During a median follow-up of 3.1 years, the median predicted risk based on IIgAN-PT was 6.5%, while 26% achieved the primary outcome and 57% showed a decline in eGFR (median annual decline of 5.6 mL/min/1.73 m^2^). This was a fourfold higher rate than that predicted by IIgAN-PT at the time of biopsy. In 2021, Barbour SJ et al. argued that IIgAN-PT was the preferred method for predicting the risk of a 50% decline in estimated eGFR or renal failure at the time of biopsy^2,11^. However, it was not known whether the prediction tool could be accurately applied after a post-biopsy observation period. Thus, they updated the tool and demonstrated that the updated version presented similar prediction performance when used two years after biopsy. Cabral et al., applying the updated IIgAN-PT one year after biopsy (n = 13), demonstrated a median predicted risk of 1.79%, although 23% achieved the primary endpoint. In the discussion, the authors point out that renal biopsy in IgAN is usually performed at more advanced stages of disease progression, in many cases with significant loss of renal function, hypertension, and persistent proteinuria, even with adequate blockade of the renin-angiotensin-aldosterone system. The authors also mentioned that late diagnosis and referrals from other centers may have contributed to the advanced stages of the disease observed at biopsy and the late initiation of immunosuppressive therapy^2^.

## CONCLUSION

Despite limitations such as small sample size, single-center design, and short follow-up, the study offers relevant clinical insights. The two main messages from the study are: (1) the prognostic tools had low ability to predict eGFR decline and progression to end-stage CKD; and (2) the treatment plans for pediatric IgAN should be individualized based on proteinuria levels, blood pressure, eGFR, histology, and response to treatment. The Oxford MEST-C score is recommended for biopsy interpretation, despite limited validation in children.

Although Barbour et al. (2021) updated the tool for pediatric patients, concluding that it can accurately predict the risk of a 30% decline in eGFR or end-stage chronic kidney disease in children with IgAN, the data presented reinforce the need to improve our knowledge of IgAN.^12^ The new clinical practice guidelines for the disease are an important source of updates and insights into early diagnosis, monitoring, and treatment.^2^ The guidelines recommend high-quality pediatric research to address key knowledge gaps, particularly regarding the efficacy of immunosuppressive therapies and long-term outcomes. These recommendations aim to standardize pediatric management of IgAN worldwide, while promoting safe, personalized, and evidence-based care. We hope that future studies will address these important issues.

## Data Availability

The data supporting the findings of this study are available with the author of the article upon reasonable request.
