# Supplementary material for: IgA nephropathy in children: how much can we trust prognostic tools?
Source: J Bras Nefrol. 2025 Sep 8;47(3):e2025E012. doi: 10.1590/2175-8239-JBN-2025-E012en (PMC12442730; doi:10.1590/2175-8239-JBN-2025-E012en)
Supplement: Translation

# Translation: Nefropatia por IgA em crianças: até que ponto podemos confiar nas ferramentas prognósticas?

**DOI:** 10.1590/2175-8239-JBN-2025-E012pt

**Authors:** Maria Goretti Moreira Guimarães Penido, Nilzete Liberato Bresolin, Oreste Ferra-Neto

**Affiliations:** 1 Hospital Santa Casa de Belo Horizonte, Centro de Nefrologia, Unidade de Nefrologia Pediátrica, Belo Horizonte, MG, Brasil.; 2 Universidade Federal de Minas Gerais, Faculdade de Medicina, Hospital das Clínicas, Unidade de Nefrologia Pediátrica, Belo Horizonte, MG, Brasil.; 3 Universidade Federal de Santa Catarina, Faculdade de Medicina, Florianópolis, SC, Brasil.; 4 Hospital Infantil Joana de Gusmão, Unidade de Nefrologia Pediátrica e Terapia Intensiva, Florianópolis, SC, Brasil.; 5 Universidade Federal do Mato Grosso do Sul, Hospital Universitário Maria Aparecida Pedrossian, Unidade de Nefrologia Pediátrica, Campo Grande, MS, Brasil.

A nefropatia por deposição de imunoglobulina A (NIgA) resulta de depósitos de complexos imunes IgA1-IgG nos glomérulos, promovendo uma cascata inflamatória que leva à injúria renal^1,2^. Trata-se da glomerulonefrite primária mais comum em todo o mundo e seu diagnóstico definitivo é feito por biópsia renal^3,4,5^.

A *International Pediatric Nephrology Association* (IPNA) publicou recomend-ações abrangentes de práticas clínicas baseadas em evidências para o diagnóstico e o manejo de crianças com NIgA. As diretrizes destacam as principais diferenças entre a NIgA pediátrica e a adulta, conforme confirmado recentemente por um amplo estudo realizado na população chinesa^2,6^. Crianças frequentemente apresentam hematúria macroscópica durante infecções (hematúria sinfaringítica), e as biópsias renais normalmente revelam uma inflamação mais ativa com menos danos crônicos em comparação aos adultos^6^. A biópsia renal é altamente recomendada em casos de hematúria persistente ou recorrente associada à proteinúria significativa (UPCR ≥ 0,5 mg/mg), proteinúria na faixa nefrótica (UPCR ≥ 2 mg/mg) ou TFGe reduzida (< 90 mL/min/1,73 m^2^). A biópsia também é necessária em pacientes com hematúria microscópica persistente e UPCR entre 0,2-0,5 mg/mg em diversas avaliações.

A doença raramente progride para o estágio 5 da doença renal crônica (DRC) na infância^7^, mas trata-se de uma doença crônica e os pacientes pediátricos apresentam risco de progressão ao longo da vida adulta, embora remissões espontâneas sejam possíveis em casos leves^8^. Assim, a NIgA pediátrica é reconhecida como uma doença que requer cuidados especializados^9^.

Os cuidados de suporte continuam sendo a base do tratamento: os bloqueadores do sistema renina-angiotensina (BSRA) são recomendados para casos de proteinúria persistente (UPCR ≥ 0,2 mg/mg), juntamente com restrição de sal na dieta e medidas relacionadas ao estilo de vida. Para crianças com DRC em estágio 2 ou superior, recomenda-se metas mais rigorosas de pressão arterial. Glicocorticoides não são recomendados rotineiramente, mas podem ser considerados em casos selecionados com proteinúria persistente, após 3-6 meses de terapia de suporte otimizada, em pacientes com proteinúria persistente ≥ 1 mg/mg ou achados de biópsia de alto risco. É importante observar que a diretriz adverte contra o uso de imunossupressores sem confirmação histológica^2^.

O acompanhamento de longo prazo é enfatizado para todos os pacientes mesmo aqueles em remissão devido ao risco de recidiva tardia e progressão para DRC^2^.

Considerando que o manejo da NIgA pediátrica ainda é controverso e apoiado por poucas evidências de alta qualidade, e que a escassez de evidências está relacionada à heterogeneidade da doença em crianças e adolescentes e sua variabilidade interétnica, o estudo de Cabral et al., publicado no Jornal Brasileiro de Nefrologia, merece algumas observações e considerações baseadas em publicações recentes.

## Resultados do Estudo de Cabral MF et al.

Cabral et al.^10^ estudaram retrospectivamente 23 crianças com NIgA. Os autores compararam os desfechos renais reais com os desfechos previstos pela *International IgAN Prediction Tool for Children* (IIgAN-PT)^11,12^. O desfecho primário foi um resultado composto de redução ≥30% na TFGe ou progressão para doença renal em estágio terminal (DRET). Durante um acompanhamento mediano de 3,1 anos, o risco mediano previsto com base na IIgAN-PT foi de 6,5%, enquanto 26% alcançaram o desfecho primário e 57% apresentaram um declínio na TFGe (declínio anual mediano de 5,6 mL/min/1,73 m^2^). Essa taxa foi quatro vezes superior à prevista pela IIgAN-PT no momento da biópsia. Em 2021, Barbour SJ et al. argumentaram que a IIgAN-PT era o método preferido para predizer o risco de um declínio de 50% na TFG estimada ou insuficiência renal no momento da biópsia^2,11^. No entanto, não se sabia se a ferramenta de predição poderia ser aplicada com precisão após um período de observação pós-biópsia. Dessa forma, eles atualizaram a ferramenta e demonstraram que a versão atualizada apresentou desempenho preditivo semelhante quando utilizada dois anos após a biópsia. Cabral et al., ao aplicarem a IIgAN-PT atualizada um ano após a biópsia (n = 13), demonstraram um risco mediano previsto de 1,79%, embora 23% tenham atingido o desfecho primário. Na discussão, os autores destacam que a biópsia renal na NIgA é geralmente realizada em estágios mais avançados da progressão da doença, em muitos casos com perda significativa da função renal, hipertensão e proteinúria persistente, mesmo com o bloqueio adequado do sistema renina-angiotensina-aldosterona. Os autores também mencionaram que o diagnóstico tardio e os encaminhamentos provenientes de outros centros podem ter contribuído para os estágios avançados da doença observados na biópsia e para o início tardio da terapia imunossupressora^2^.

## Conclusão

Apesar das limitações, como o pequeno tamanho da amostra, o desenho de centro único e o curto período de acompanhamento, o estudo oferece percepções clínicas relevantes. As duas principais mensagens do estudo são: (1) as ferramentas prognósticas apresentaram baixa capacidade de prever o declínio da TFGe e a progressão para DRC em estágio terminal; e (2) os planos de tratamento para NIgA pediátrica devem ser individualizados com base nos níveis de proteinúria, pressão arterial, TFGe, histologia e resposta ao tratamento. O escore Oxford MEST-C é recomendado para a interpretação da biópsia, apesar da validação limitada em crianças.

Embora Barbour et al. (2021) tenham atualizado a ferramenta para pacientes pediátricos, concluindo que ela pode prever com precisão o risco de um declínio de 30% na TFGe ou doença renal crônica terminal em crianças com NIgA, os dados apresentados reforçam a necessidade de aprimorar nosso conhecimento sobre a NIgA.^12^ As novas diretrizes de prática clínica para a doença constituem uma fonte importante de atualizações e percepções sobre diagnóstico precoce, monitoramento e tratamento.^2^ As diretrizes recomendam a realização de pesquisas pediátricas de alta qualidade para abordar as principais lacunas de conhecimento, particularmente em relação à eficácia das terapias imunossupressoras e aos desfechos de longo prazo. Essas recomendações visam padronizar o manejo pediátrico da NIgA em nível global e, ao mesmo tempo, promover um cuidado seguro, personalizado e baseado em evidências. Esperamos que estudos futuros abordem essas importantes questões.

## Disponibilidade de Dados

Os dados que embasam os achados deste estudo estão dispsoníveis com o autor do artigo, mediante solicitação razoável.
